# Neurological and neuroimaging sequelae of lightning injury: a retrospective study and literature review

**DOI:** 10.1186/s42466-026-00523-2

**Published:** 2026-09-14

**Authors:** Tim Steinberg, Carsten Lukas, Berthold Schalke, Christina Wendl, Ralf Linker, Bernhard Neumann, Ingo Kleiter

**Affiliations:** 1 Department of Neurology, University Medical Center Regensburg, Regensburg, Germany; 2 Department of Neurology and Neurophysiology, University Medical Center Augsburg, Augsburg, Germany; 3 Institute of Neuroradiology, St. Josef-Hospital, Ruhr University Bochum, Bochum, Germany; 4 Department of Neuroradiology, University Medical Center Regensburg, Regensburg, Germany; 5 Department of Neurology, Donau-Isar-Clinic Deggendorf, Medical Campus Niederbayern, Deggendorf, Germany; 6 Department of Neurology, St. Josef-Hospital, Ruhr University Bochum, Bochum, Germany; 7 Marianne-Strauß-Klinik, Berg, Germany

**Keywords:** Lightning injury, Intracerebral bleeding, Myelopathy, Climate change

## Abstract

**Background:**

Lightning injuries (LI) can affect multiple organs, including the peripheral and central nervous system (CNS). Neuroimaging studies in LI are scarce, and it is unknown whether off-target changes in the CNS occur.

**Methods:**

Retrospective review of patients who were treated in two neurology tertiary referral centers for sequelae of LI between 2004-2024 and had magnetic resonance imaging (MRI) or computed tomography (CT) of the CNS. A narrative literature search was performed to identify other studies on neuroimaging after LI.

**Results:**

Of 31 patients (39% female, median age 38 years), 30 had cranial neuroimaging (28 had cranial MRI, five cranial CT), and 14 spinal MRI. The median delay between LI and first neuroimaging was 67 days. Cranial MRI or CT showed two cases of brain edema, one accompanied by subarachnoid bleeding, one case of laminar cortical necrosis after resuscitation, and one case with hyperintensities in the hippocampi. 14 patients with cranial MRI had unspecific white matter lesions. In six of 14 patients who received a spinal MRI, intramedullary spinal cord lesions were found. A review of 54 published patients revealed pathological neuroimaging in 24 (44%) cases. Main findings were hypoxic encephalopathy after cardiac arrest, intracerebral bleeding, cerebral ischemia, brain edema and subarachnoid bleeding.

*Interpretation:* Typical neuroimaging findings after LI include brain edema, intracerebral or subarachnoid bleeding, ischemia, hypoxic encephalopathy, and intramedullary spinal cord lesions. Moreover, brain white matter lesions are common, which are of unknown significance, since clinical correlates are rare and pre-existing pathologies often cannot be ruled out.

**Supplementary Information:**

The online version contains supplementary material available at https://doi.org/10.1186/s42466-026-00523-2.

## Introduction

Lightning strike injuries (LI) are rare events. An average of 64 annual deaths were reported throughout Europe in the period 2001-2020 by the European Severe Storms Laboratory, and an average of four deaths per year were reported in Germany[1, 2]. At least five major types of LI-induced damage can be distinguished: direct strike (where the patient is hit directly by lightning), contact voltage (where the patient has direct contact with an object hit by lightning), side splash (where a nearby object is hit by lightning and a part of the energy flashes over to the patient), ground current effect (the lightning hits the ground and a potential difference between two limbs of the patient attached to the ground leads to a current), wire mediated lightning (the lightning hits a power supply line, telephone wire or a via ferrata wire and the energy is hence conducted to the patient), and blast injuries [3]. Despite the extremely high energies involved, only about 20% of patients directly die due to a lightning accident [4]. Both peripheral and central nervous system disorders resulting from LI have been reported. Those include intracerebral bleeding [5], subarachnoid bleeding [6], cerebral ischemia [7], brain edema [8], hypoxic brain damage due to cardiac arrest [9], Guillain-Barré syndrome [10], plexus neuritis [11], keraunoparalysis [12, 13], neuropsychological deficits [14], and more. Cherington classified these into four groups: acute transient (e.g. loss of consciousness), acute permanent (e.g. intracerebral bleeding), subacute permanent (e.g. neuropsychological deficits) and secondary, for example due to the compression wave or physical trauma [15]. Accordingly, a multitude of different pathologies have been identified through neuroimaging of those patients. The aim of this study was to provide an overview of the possible findings in neuroimaging and to define the proportion of patients exhibiting pathological changes following LI. We therefore describe 31 cases from our database and review existing literature on LI and neuroimaging.

## Methods

### Study design and patient selection

We did a retrospective chart review of patients who attended the Neurology Departments of the University of Regensburg and the St. Josef Hospital, Ruhr-University Bochum in the years 2004-2024. We identified all patients with a history of a LI and included all patients who had received neuroimaging (MRI or CT) of the brain or MRI of the spinal cord as part of their diagnostic workup. Data on baseline demographics, clinical information and comorbidities were obtained through medical charts and institutional databases.

### Data acquisition – neuroimaging

Of the 31 included patients, 19 received neuroimaging at the University of Regensburg. MRI imaging was done with Siemens Magnetom Sonata 1.5 Tesla and Siemens Magnetom Aera 1.5 Tesla MRI scanners. In the remaining patients, both MRI and CT scans were supplied by external radiologists and images from electronic data sources were reviewed. Neuroimaging studies were evaluated by experienced neurologists (T.S., I.K.) and neuroradiologists (C.L, C.W.).

### Review of literature

A narrative literature research using pubmed.org was done by one investigator (T.S.). The following search terms were employed: "lightning", "lightning strike", "lightning injury", "lightning sequelae", "magnetic resonance imaging AND lightning strike", "computed tomography AND lightning strike", "radiology AND lightning strike". The inclusion criteria were LI and the presence of neuroimaging studies. Patients with electrical accidents other than LI were excluded from the study. The time frame selected for the study spanned from 01.01.1990 to 01.04.2024, encompassing the period during which modern computed tomography (CT) and magnetic resonance imaging (MRI) scanners were widely utilized. Baseline information (age, sex), data on imaging and clinical data (type of injury, neurological symptoms) were extracted from the publications.

### Ethical approval

This study was performed in line with the principles of the Declaration of Helsinki. The ethics committee at the University of Regensburg approved the study (#24-3839-104).

### Consent to publish

Written informed consent-to-disclosure was obtained from the three patients described in detail in the section illustrative cases. For the remaining patients in our case series, data have been anonymized by removing potentially identifying characteristics, such as age and sex.

## Results

### Study population and clinical findings

We identified 48 patients who were treated in our clinic for neurological sequelae of LI between 2004 and 2024. Of those, 31 patients (65%) had received imaging of the brain or spinal cord and were included for further analysis. 12 patients were female and the median age was 38 years (interquartile range (IQR) 27 years). The median time between LI and first presentation in our clinic was 308 days (IQR 1038 days), and the median delay between LI and first neuroimaging was 67 days (IQR 715 days). In 28 cases cranial MRI was performed, MRI of the spinal cord was done in 14 cases and cranial CT in five cases.

Clinical examination revealed sensory deficits in 21 cases (68%) and persisting motor impairments (excluding acute keraunoparalysis) in 15 cases (48%). Neuropsychological deficits were diagnosed in 12 cases (39%) using a neuropsychological test battery. Furthermore, in 14 cases (45%) psychiatric disorders, including depression, post-traumatic stress disorder, anxiety, and specific phobia (i.e. for thunderstorms) emerged following LI. For more detailed information on clinical presentations and neuroimaging findings see Table 1.

**Table 1:** Summary of clinical and radiological findings of 31 patients after lightning strike injury

Case	Type of LI	Radiological findings (interval fom LI)	Clinical presentation (interval fom LI)	Medical history
1	a or b	cMRI (2 years): unspecific left parietal white matter lesions, diffuse supra- and infratentorial atrophy.	Acute: exhaustion, lack of concentration, gait problems, visual pseudo hallucinations. Clinic (2 years): horizontal diplopia when looking to the left side, gait ataxia, slight ataxia of the left arm, hypalgesia, hypesthesia and hypothermesthesia below Th10 on the left, depression, neuropsychological deficits.	Migraine
2	a	cCT (3 hours): bilateral brain edema of the occipital lobe. cMRI (6 days): bilateral occipital subarachnoid bleeding.	Acute: loss of consciousness, disorientation, persistent inferior visual field loss. Clinic (20 days): small area of hypesthesia beneath the left eye, inferior visual field loss, neuropsychological deficits, depression.	No relevant previous diagnoses
3	c	cMRI (2 months): two unspecific white matter lesions in the semioval center on the left	Acute: paroxysmal parietal headache, depression. Clinic (31 days): distal thermhypesthesia, hypesthesia and pallhypesthesia of both legs, bilateral Horner's syndrome, depression, anxiety.	Colorectal cancer, hypertension
4	c	cMRI (3 days): two subcortical lesions in the right frontal lobe and parasagittal on the left. sMRI (9 days): no lesions sMRI (1 year) hyperintense lesion C5/C6. cMRI (2.7 years): detection of two lesions, each measuring 1 cm, located in the right frontolateral white matter and at the left cortical margin. sMRI (2.7 days): additional lesions C1/C2.	Acute: keraunoparalysis Clinic (1 day): plantar hypesthesia of both feet and the left forehead, positive Lhermitte's phenomenon, premature ejaculation.	Traumatic brain injury left frontal lobe 6 years earlier, nicotine use
5	b	cCT (few hours): normal	Acute: keraunoparalysis, loss of consciousness, hypesthesia of both feet. Clinic (1 year): distal symmetric hypesthesia, hypalgesia, hypothermesthesia, pallhypesthesia, neuropsychological deficits.	No relevant previous diagnoses
6	a or b	cMRI (24 days): normal sMRI (20 days): normal	Acute: CPR. After extubation accommodation difficulties, gait ataxia, hypesthesia of both hands and lower legs, neuropsychological deficits and depression. Clinic (32 days): gaze-evoked nystagmus in all directions of view, gait ataxia with tendency to fall to the left, distal asymmetric (left>right) hypesthesia and pallhypesthesia of both legs, neuropsychological deficits.	No relevant previous diagnoses
7	c	sMRI (3.4 years): normal sMRI (8.2 years): intramedullary lesion C2-C4. cMRI (15.6 years): normal	Acute: hypesthesia digiti III/IV on the left. Ascending hypesthesia of the limbs, gait ataxia. Clinic (15.5 years): distal symmetric hypesthesia, areflexia, slight distal paresis of both legs, gait ataxia, skew deviation, neuropsychological deficits.	Previous traumatic brain injury
8	b or c	MRI cervical spine (2 months): normal	Acute: loss of consciousness, keraunoparalysis of both hands. Clinic (1.1 years): neuropsychological deficits.	Bilateral disc herniation at L4/5 one year earlier
9	b	sMRI (1.7 years): intramedullary lesion C1-C5 affecting the posterior funiculus on both sides. cMRI (2 years): normal sMRI (2.3 years): no change sMRI (3.3years): no change	Acute: loss of consciousness, keraunoparalysis, persisting numbness in both hands and feet, gait ataxia. Ascending hypesthesia up to cervical level, anhidrosis of both hands. Clinic (2.4 years): hypesthesia, hypalgesia, hypothermesthesia, pallanesthesia below level L1, astereognosia, slight hemiataxia on the left, Babinski's sign positive on the right > left. Neuropsychological deficits.	History of nicotine use
10	c	cCT (9 months): normal	Acute: Transient hypesthesia of both hands. Clinic (5 months): slight hypesthesia below L1 level. Neuropsychological deficits.	No relevant previous diagnoses
11	a	cMRI (16 days): unspecific frontal subcortical lesions on both sides.	Acute: CPR. After extubation hallucinations, central scotoma, paraparesis of the legs. Clinic (2.9 years): gait ataxia, areflexia, moderate distal and symmetrical sensory impairment for all qualities, plantar anhidrosis of both feet, neuropsychological deficits.	History of nicotine use, hypertension
12	b or c or d	cMRI & MRI cervical spine (14 days): normal cMRI (9.5 years): normal sMRI (9.5 years): hyperintense intramedullary lesion in the lumbar cord.	Acute: CPR. After extubation gait ataxia, unspecific body pain and allodynia, transient inability to speak formerly known foreign languages. Clinic (9.2 years): patchy hypalgesia, hypesthesia and pallhypesthesia of arms and feet, distal weakness of the hands (MRC 4/5) with atrophy of small hand muscles, areflexia of the arms, gait ataxia, specific phobia, neuropsychological deficits.	No relevant previous diagnoses
13	d	sMRI (1 month) & cMRI (1.3 years): normal	Acute: keraunoparalysis. Clinic (1.5 years): hypesthesia and hypalgesia of ophthalmic nerve on the left, diminished fine motor skills of the right hand, hypesthesia, pallhypesthesia and thermhypesthesia of both legs left>right, weakness of both legs (MRC 4/5), gait ataxia, specific phobia, neuropsychological deficits.	nicotine use, hypertension, diabetes mellitus
14	b or c	cMRI (11.3 years): normal	Acute: keraunoparalysis. Clinic (17.5 years): hemihypesthesia and hemihypalgesia on the left, hemiataxia on the left, anxiety disorder.	No relevant previous diagnoses
15	d	cMRI (5 months): unspecific juxtacortical lesions, mainly on the left side.	Acute: keraunoparalysis, recurrent transient paresthesia of the right face. Clinic (7 months): hypesthesia and hypalgesia of the face on the right side, akinetopsia, impaired corneal reflex on the right eye, diminished fine motor skills of the right hand, minor neuropsychological deficits.	No relevant previous diagnoses
16	b or c	cMRI (10 days): hyperintense cortical and subcortical lesions localized in the frontal, occipital and parietooccipital lobes sMRI (18 days): gadolinium-positive cauda equina and nerve fibers	Acute: CPR, after extubation paraplegia and areflexia of lower extremities. Hypesthesia, pallhypesthesia and hyperpathia of both legs.	No relevant previous diagnoses
17	a	cMRI (3 days): normal cMRI (31 days): normal	Acute: loss of consciousness, keraunoparalysis of both legs and left arm. Secondary CPR. After release from the hospital posttraumatic stress disorder, depression. Clinic (10 months): neuropsychological deficits.	No relevant previous diagnoses
18	b or d	cMRI (1.1 years): normal sMRI (1.5 years): minor disc protrusions without stenosis of the spinal canal. Otherwise unremarkable.	Acute: loss of consciousness. Clinic (1.2 years): right-sided facial paresis, intermittent, involuntary movements of the left leg and fasciculations. Proximally pronounced tetraparesis. Finger-to-nose test and heel-to-knee test on the left side dysmetric. Cognitive deficits.	Hypertension
19	d	cMRI (approx. 3 years): supratentorial small, nonspecific periventricular lesions on both sides. Small pineal cyst without evidence of cerebrospinal fluid outflow obstruction. Otherwise unremarkable.	Acute: right-sided hemiparesis, recurrent cramps on the right side. Clinic (3 years): gaze-evoked nystagmus in both directions, right-sided falling tendency, minimal right arm paresis, gait instability, subtle dysmetria on the right during the finger-to-nose and heel-to-knee tests. Cognitive deficits.	No relevant prior diagnoses
20	a	cMRI (6 months): unremarkable, except for a few small, nonspecific periventricular lesions.	Acute: CPR. Dysarthria, right-sided ataxia, organic psychosis syndrome and cognitive deficits. Clinic (6 months): mild weakness of the left arm and right-sided hemihypesthesia. Cognitive deficits.	Hypertension
21	a or b	cMRI (4 months): unremarkable, except for a few small, nonspecific periventricular lesions.	Acute: disorientation, right-sided persistent headache, scalp hypersensitivity, intermittent gait instability, dizziness and cognitive deficits. Clinic (4 months): broad-based gait pattern and distal pallhypesthesia of the legs.	No relevant previous diagnoses
22	probably c	cCT (initially): ventricular asymmetry favoring the right side, with evidence of right-sided swelling, without signs of cerebrospinal fluid (CSF) outflow obstruction. cMRI (day 1): supratentorial small, nonspecific periventricular lesions on both sides.	Acute: Initial presentation in our emergency department. Mild headache at first, no focal neurological deficits. Neuropsychological deficits.	No relevant previous diagnoses
23	b or d	cMRI (2 months): unremarkable, except for a few small, nonspecific periventricular lesions. sMRI (2 months): unremarkable, except for a few disc protrusions.	Acute: CPR and long-lasting intensive care unit treatment. Clinic (40 days): right-sided tetraparesis, dysarthria, atrophy of distal musculature, areflexia, ataxic finger-to-nose test.	No relevant previous diagnoses
24	e	cCT (13 days): normal cMRI (date unclear): normal	Acute: pain in both arms and the left foot, dizziness, restlessness, headaches, and sensitivity to light and sound. Clinic (3.2 years): dizziness and paresthesia in the soles of the feet.	No relevant previous diagnoses
25	c	cMRI (2.1 years): supratentorial small, nonspecific periventricular lesions on both sides. sMRI (2.1 years): normal	Acute: loss of consciousness, keraunoparalysis, cognitive deficits. Clinic (2.1 years): unremarkable.	Hypertension
26	NA	cMRI (12 years): supratentorial small, nonspecific periventricular lesions on both sides.	Acute: hyperhidrosis, sensation of heat in the feet, hypesthesia and paresthesia of the feet, cognitive deficits, dizziness, and persistent headaches. Clinic (12 years): ataxia in the finger-to-nose test on the right side.	No relevant previous diagnoses
27	c	cMRI (2 days): supratentorial small, nonspecific periventricular lesions on both sides. cMRI (1.5 years): increasing number of white matter lesions.	Clinic (2 days): paresthesias on the right side of the face, and pre-existing, patchy hypesthesia on the left side of the body.	No relevant previous diagnoses
28	NA	cMRI (2 months): progressive widening of the internal and external cerebrospinal fluid spaces, indicative of diffuse atrophy. Laminar cortical necrosis bilaterally in the occipital lobes, as well as T2-FLAIR hyperintensities in the basal ganglia bilaterally, consistent with residuals of a prior basal ganglia infarction. sMRI (2 months): symmetrical lesion in the conus medullaris.	Acute: CPR. Right sided brachiofacial hemiparesis. Clinic (2 months): anisocoria (left > right), dysarthria, right-sided facial paresis, pronation during the arm-holding test on the right, mild paraparesis of the legs, neuropsychological deficits.	No relevant previous diagnoses
29	d	cMRI (8 days): apart from a mild prominence of the external cerebrospinal fluid spaces in the frontoparietal region, unremarkable. sMRI (11 days): normal	Acute: asymptomatic. Clinic (7 days): neuropsychological deficits.	No relevant previous diagnoses
30	NA	cMRI (13 years): diffuse atrophy, including the hippocampi, with T2-weighted signal hyperintensities.	Acute: spastic tetraparesis, gait ataxia, dysarthria, cognitive symptoms and epileptic seizures. Clinic (13 years): dysarthria, no paresis, but increased muscle tone with a predominance in the right side and lower limbs; gait ataxia with a tendency to fall to the right; dysmetria on both sides in the finger-to-nose and heel-to-shin tests; distal paresthesias (particularly in the right upper limb); distal pallhypesthesia.	No relevant previous diagnoses
31	b or c	cMRI (3 months): normal sMRI (3 months): normal	Acute: CPR, right-sided facial palsy and a selective inability to speak a foreign language lasting a few hours, scotoma in the upper right visual field of the right eye. Clinic (2 months): tongue deviation to the left, mild gait ataxia during challenging gait tests, and dysesthesias of the arms and feet.	No relevant previous diagnoses

cMRI = cranial magnetic resonance imaging; sMRI = spinal magnetic resonance imaging; CPR: cardiopulmonary resuscitation, LI: Lightning strike injury, MRC: Medical Research Council, ROSC: Return of spontaneous circulation, type of LI: a = direct strike, b = side splash, c = ground current, d = contact voltage, e = wire mediated, NA = not available.

### Neuroimaging results

Cranial imaging was done in 30 patients, 25 had exclusively MRI, two exclusively CT, and three both MRI and CT (Table 1). In this group, two cases of brain edema, one accompanied by subarachnoid bleeding were identified, as well as one case with laminar cortical necrosis and basal ganglia infarction after resuscitation and one case showing hyperintensities of both hippocampi 13 years after LI. Unspecific white matter lesions were observed in 14 of 28 patients with cranial MRI (50%). Nine of these patients exhibited cognitive deficits. Additional neurological findings included various sensory and motor signs. However, in retrospect, central nervous system damage could not be reliably attributed to specific CNS white matter lesions and distinguished from the frequently occurring peripheral nerve involvement, since only some patients have received electrophysiologic workup. Cardiovascular risk factors were present in five of 14 cases with white matter lesions (36%). 12 of 30 patients (40%) who had brain scans had no pathological findings; however, all of them were complaining about various, sometimes transient neurological symptoms and had neurological findings, mostly affecting the peripheral nervous system or showing neuropsychological deficits.

Spinal cord imaging by MRI was done in 14 patients. Interestingly, in six patients (43%) intramedullary lesions were observed. These lesions were found in the cervical spine (one case in C1/2 and C5/6, one case in C2-C4, and one case in the posterior funiculus of C1-C5) and the lumbar cord or cauda equina (three cases). Specific symptoms corresponding to these lesions were often masked by symptoms originating from damage to the brain or peripheral nervous system. Three patients presented with paraparesis of the lower limbs, one exhibited gait ataxia, sensory deficits and bilaterally positive Babinski sign, and one reported premature ejaculation.

### Illustrative cases

Case #2: 24-year-old woman with visual field loss and occipital subarachnoid bleeding

**Fig. 1:**
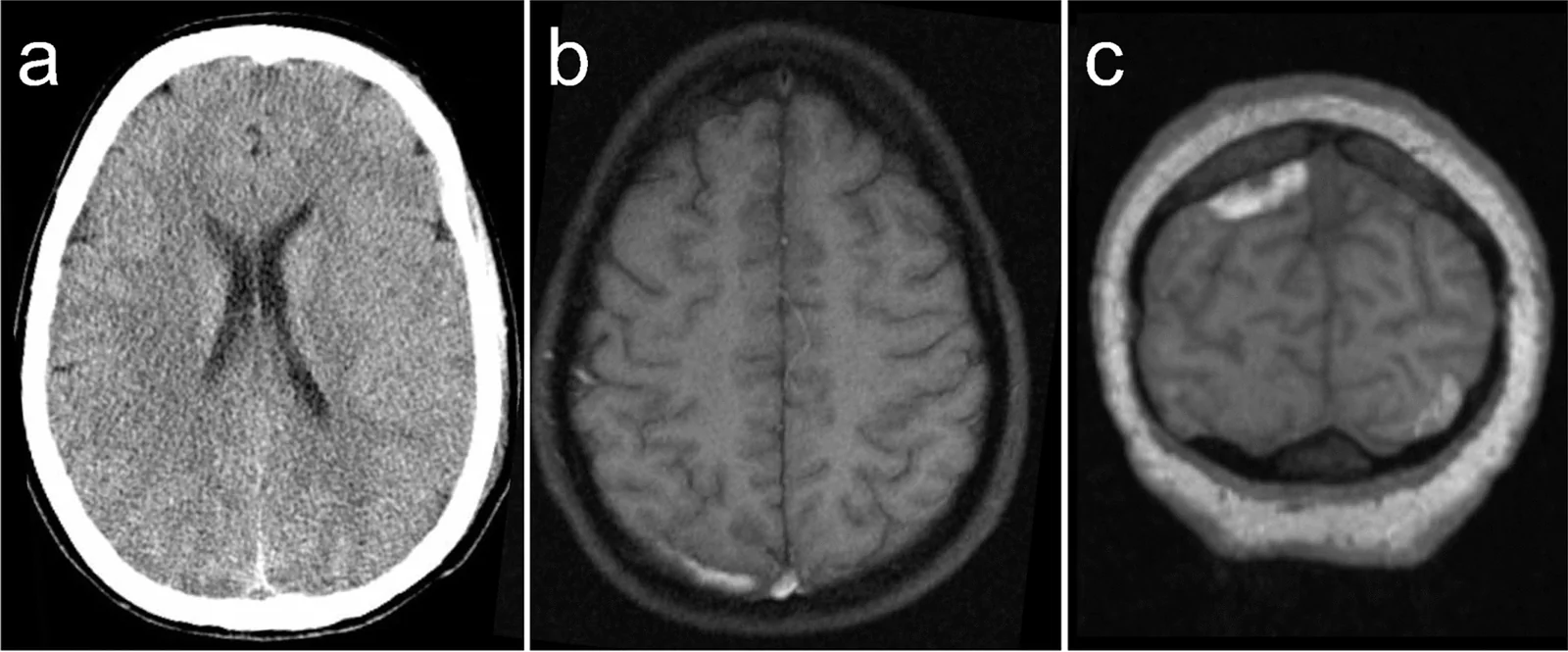
Neuroimaging case #2. (**a**) CT scan performed three hours after the injury revealing slight occipital brain edema. Axial (**b**) and coronal section at level of the occipital and parietal lobe (**c**) T1-weighted images conducted six days after LI demonstrated occipital subarachnoid bleeding[14]

A 24-year-old female patient was directly hit by a lightning strike to the head while climbing in the mountains [14]. This resulted in a transient loss of consciousness for a few seconds, after which the patient reported a complete loss of vision. The initial cranial CT scan revealed slight bilateral occipital oedema, with no further pathologies. Subsequent MRI scans, performed six days and eleven days after the initial injury, revealed occipital subarachnoid bleeding (Fig. 1). The patient reported a scotoma in the central downward visual field in both eyes, as well as a bifrontal headache. Although the brain MRI four weeks after the injury was normal, the scotoma persisted, and the patient subsequently developed post-traumatic stress disorder.

Case #9: 50-year-old man with a spinal lesion and secondary deterioration

**Fig. 2:**
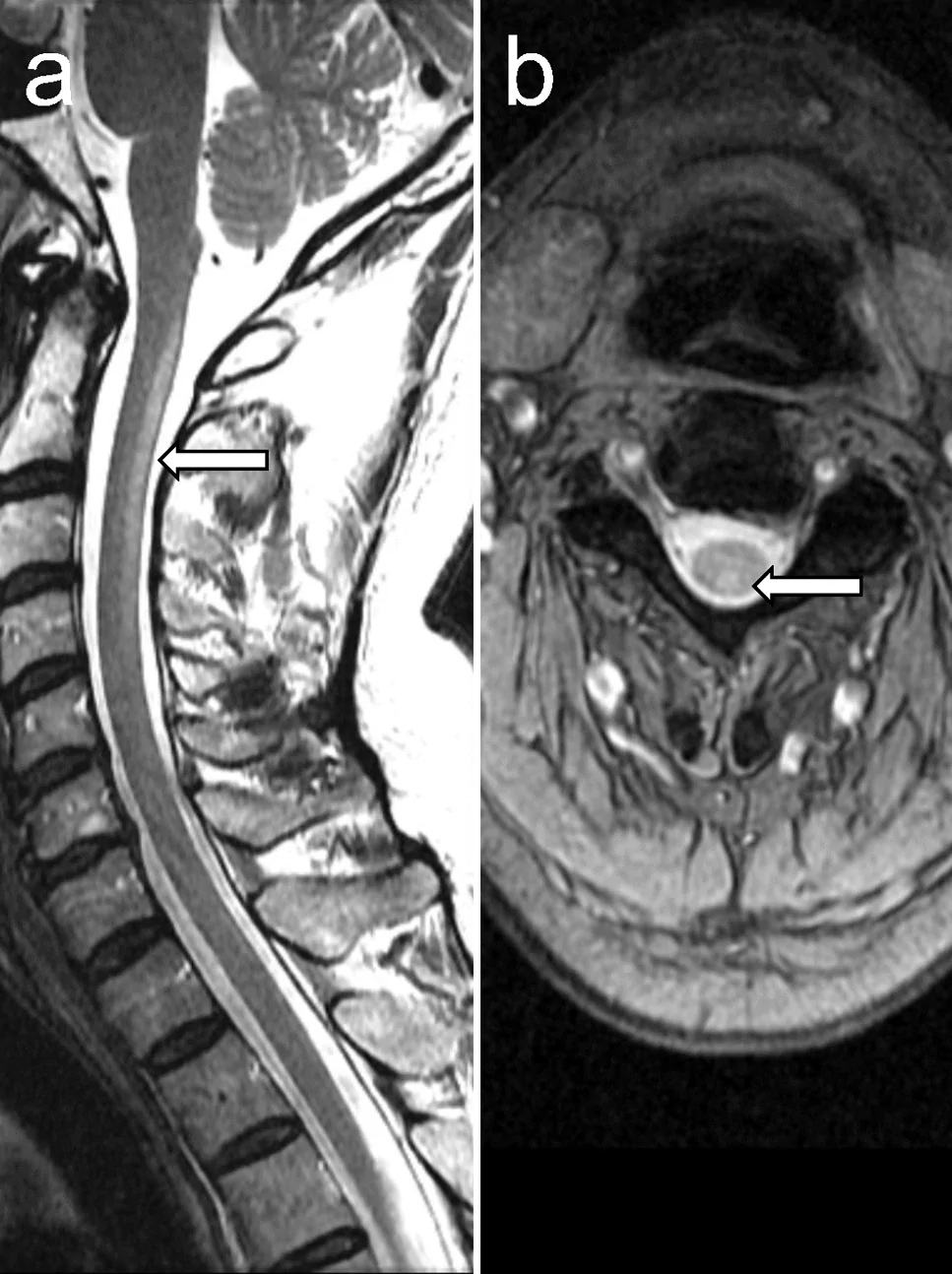
Neuroimaging case #9. Sagittal (**a**) and axial (**b**) T2-weighted image 21 months after LI showed a hyperintense lesion of the posterior funiculus ranging from C1 to 5

A 50-year-old man sustained a side splash while hiking in the mountains in bad weather conditions. Immediately after the injury, the patient lost consciousness for approximately 20 to 30 minutes. The patient had sensorimotor tetraplegia, which resolved incompletely within 24 hours and was diagnosed as keraunoparalysis. During the following weeks, the patient exhibited gait ataxia, sensory deficits in both feet, and abdominal and cervical pain. Sixteen months after the injury, the patient developed progressive, ascending hypesthesia and paresthesia, as well as weakness of the lower limbs. At the initial presentation at our clinic, 29 months after the injury, the patient demonstrated gait ataxia, mild paresis of both lower limbs, with a positive Babinski sign, hypesthesia below the L1 level, astereognosia, and pallhypesthesia in both legs. Neurophysiological studies demonstrated a latency delay in transcranial magnetic stimulation to the legs and loss of somatosensory evoked potentials in both legs. Cranial MRI (24 months after the injury) revealed no pathology, while spinal MRI (21 and 27 months after the injury) revealed a multisegmental, T2-hyperintense lesion in the posterior funiculus from C1 to C5 (Fig. 2). Following intensive physiotherapy and symptomatic medical treatment, there was improvement in both motor functions and paresthesias. A subsequent spinal MRI 39 months after the injury showed persistent evidence of the spinal cord lesion, with no signs of progression or new lesions.

Case #12: 32-year-old woman with gait ataxia and lower spinal cord lesion

**Fig. 3:**
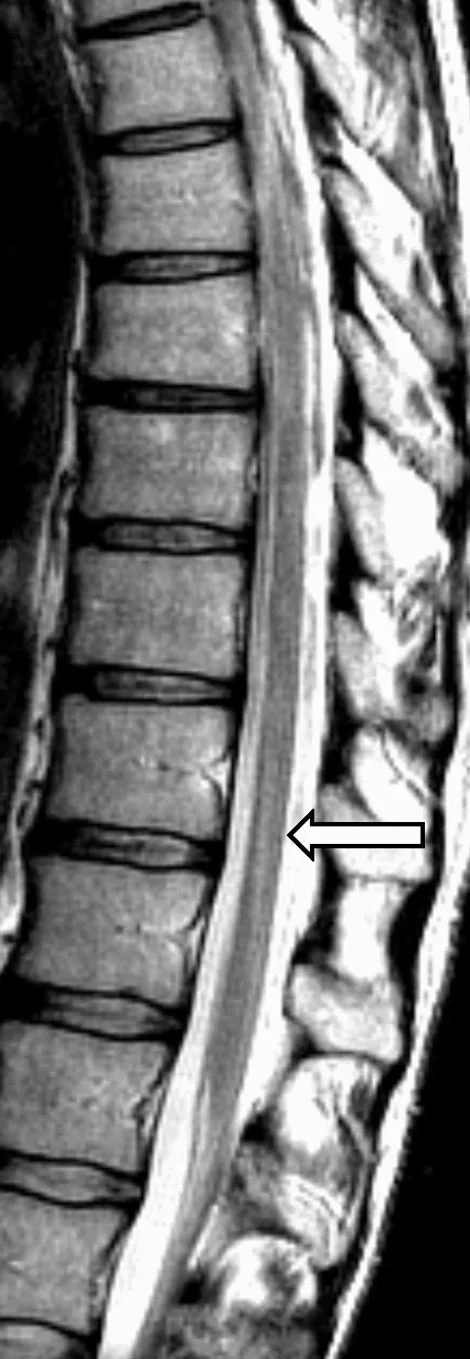
Neuroimaging case #12. Spinal MRI scan 9.5 years after LI. Sagittal T2 weighted sequence showing hyperintense lesion in the lumbar cord (arrow)

A 32-year-old woman was hit by a direct or a contact strike as she leaned against the car with her arm outstretched. Following an immediate loss of consciousness, cardiopulmonary resuscitation was performed for a duration of forty minutes. Initial MRI scans of the neurocranium and cervical spine revealed no abnormalities. During the following months after discharge from the hospital, the patient showed gait disturbance, unspecific pain of thorax, arms, head and teeth with allodynia, as well as a loss of her previously acquired linguistic skills. On presentation at our center nine years after the LI, the patient complained of persistent thoracic pain and neurological examination revealed hypesthesia C5-C8 on the right side, hypalgesia on both hands and feet, pallhypesthesia of both hands, areflexia, distal paresis of both upper extremities with atrophy of small hand muscles, and bradydiadochokinesis of both hands as well as gait ataxia. Electrophysiological investigations revealed signs of small-fiber polyneuropathy, and spinal MRI showed subtle hyperintensities in the lower spinal cord (Fig. 3).

Cases #4, #16, #28

**Fig. 4:**
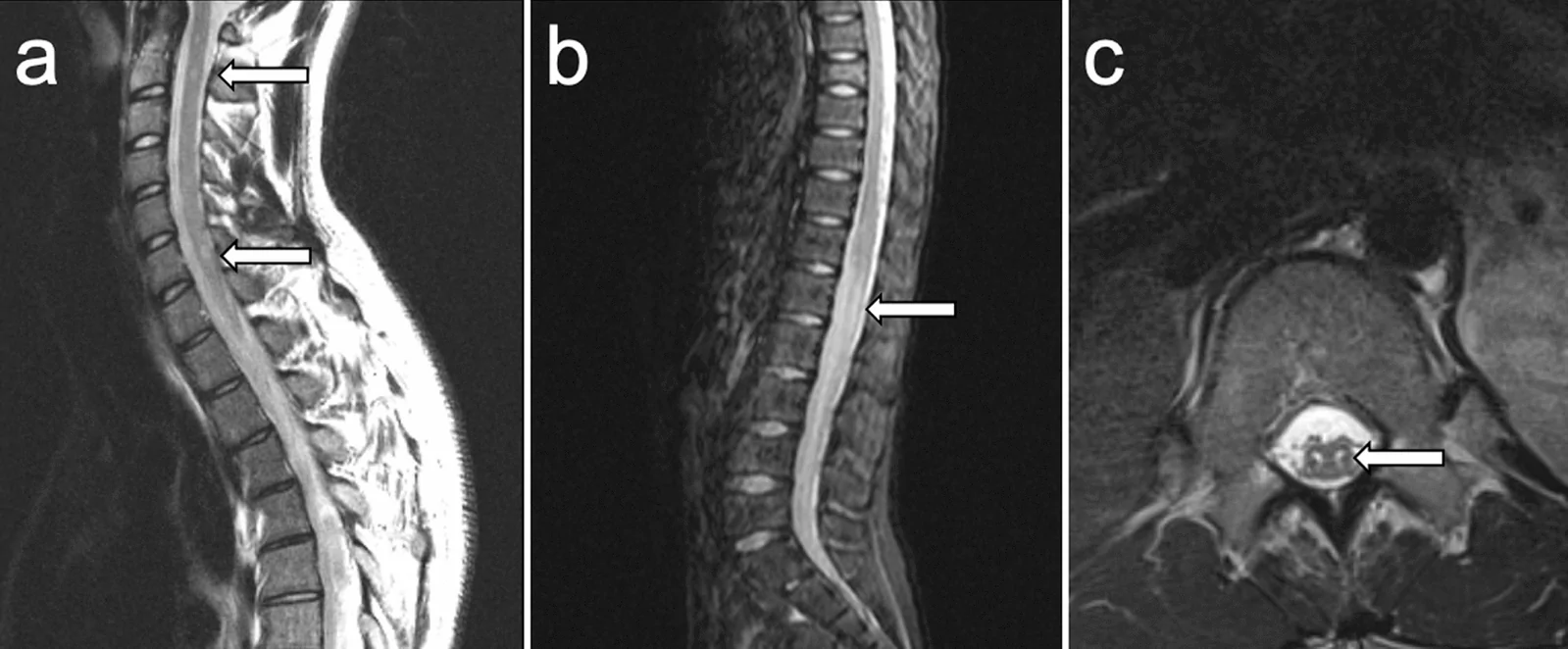
Neuroimaging findings demonstrating spinal lesions in three additional cases. (**a**) Case #4: Sagittal T2-weighted MRI obtained 1 year after LI, showing a hyperintense lesion at the C5–C6 level. (**b**) Case #16: Sagittal T2-weighted MRI obtained 18 days after LI, demonstrating contrast enhancement of the cauda equina and nerve roots. (**c**) Case #28: Axial (transverse) T2-weighted MRI obtained 70 days after LI, showing a symmetrical lesion of the conus medullaris

Beyond cases #9 and #12 we found four more cases showing spinal lesions after LI in our cohort. As this is an exceptional finding and rarely reported in literature, we provide imaging data of three of those four cases (Fig 4). In one case, imaging data was no longer available at the time of publication.

### Review of literature

Of 211 articles meeting the predefined search terms and time frame, 46 articles reporting a total of 54 cases were included. 81% of patients were male, the median age was 27 years (IQR 27 years) and the median time between injury and first imaging was one day (IQR 4 days). 49 cases received cranial CT or MRI and 15 patients received MRI of the neck or spine. In 30 cases (56%) no lesions were found on neuroimaging of the brain and/or spinal cord. In cases receiving imaging of the brain (49), common findings included intracerebral bleeding (5/49, 10%), hypoxic encephalopathy due to cardiac arrest (7/49, 14%), unspecific white matter lesions (3/49, 6%), subarachnoid bleeding (3/49, 6%), edema without a history of cardiac arrest (3/49, 6%), and cerebral ischemia (2/49, 4%). Intracerebral bleedings were predominantly localized to the basal ganglia and thalamus. In two of 15 cases with spinal MRI (13%), spinal cord lesions and corresponding neurological deficits were reported. Five of 54 cases resulted in death due to LI, three cases due to hypoxic encephalopathy, one case due to generalized brain edema, and one case due to cerebellar infarction with mass effect. Detailed information on each case is presented in Supplementary Table 1.

## Discussion

Lightning injuries are rare, but their consequences for neuronal tissue can be severe. In this study, we assessed 31 cases and conducted a comprehensive review of the literature on neuroimaging in LI survivors. Our findings revealed two key insights. Firstly, while the presence of pathological findings in neuroimaging after LI is common, a distinctive diagnostic pattern remains absent. A wide range of CNS damages was observed, including brain edema, intracranial bleeding, subarachnoid bleeding, ischemia, white matter lesions, spinal cord lesions, and post-hypoxic encephalopathy after resuscitation. Secondly, we observed that approximately 40% of the cases exhibited no acute or subacute findings on neuroimaging, despite presenting with various neurological or neuropsychological symptoms. Notably, alternative causes of the neuroimaging findings were not systematically excluded in every case. For example, some white matter lesions may be attributable to microangiopathic changes or neuroinflammatory diseases. This limitation should be kept in mind when interpreting our findings.

Clinical and neuroimaging studies have identified various sequelae and findings in the aftermath of LI (see Supplementary Table 1). Cherington *et al* proposed the following categories of clinical findings after LI according to the time of onset and progression: acute transient, acute permanent, subacute permanent, and secondary to other causes [15]. Acute transient disorders manifest as loss of consciousness, retrograde amnesia and keraunoparalysis, which typically do not have any morphological representation [12]. Of the acute permanent lesions, which may lead to long lasting deficits intracerebral bleeding, subarachnoid bleeding, ischemia and brain edema are most common and often already apparent in cranial CT, [16]. Fractures of the skull have been documented in single patients after LI [17]. The subgroup of subacute permanent injuries encompasses a wide range of conditions, including Guillain-Barre syndrome [10], neuropsychological deficits, posttraumatic stress disorder [18], and spinal cord lesions [19]. The most common morphological findings among patients with secondary diseases are hypoxic encephalopathy due to cardiac arrest and sinus thrombosis [20].

Interestingly, it appears that there is a subgroup of patients, who develop subacute myelopathy and/or radiculopathy after a LI, as shown in case #9. Our case series revealed more cases with spinal cord lesions (6/14 cases, 43%) as were found in the literature review (2/15 cases, 13%). This discrepancy may be attributed to the absence of specific and/or often mild clinical symptoms in most cases, which does not necessitate spinal imaging. It is important to acknowledge that most of the radiological findings in spinal MRI in our study and literature cases were found years after the LI, making it difficult to prove direct causality. Neuroinflammatory diseases, such as multiple sclerosis and neuromyelitis optica spectrum disorders, were not systematically ruled out in our cases or in the cases presented in literature. Furthermore, it has been postulated that electrical injury may act as a trigger for CNS autoimmunity [21].

Case #2 illustrates the direct correlation between the localization of the LI, its morphological characteristics, and clinical manifestations. The patient sustained a direct impact to the occipital region of the brain, resulting in bilateral occipital brain edema, subarachnoid bleeding, and visual field loss.

Case #16 of our study illustrates an acute keraunoparalysis with subsequent hyperintense lesions of the cauda equina, detected 18 days after injury. Keraunoparalysis is discussed to be a result of current-induced vasospasms of spinal vessels [13]. It is arguable, that if ongoing long enough, a permanent damage to the spinal cord could develop. Secondly, the electric current caused by a lightning strike could be readily conducted by the spinal cord, thereby directly causing damage.

Our clinical cohort primarily reflects subacute neuroimaging findings, as the median interval between lightning injury (LI) and the first neuroimaging examination was 67 days. In contrast, the cases identified in our literature review predominantly represented the acute phase, with a median interval of only one day between LI and first imaging. Accordingly, acute pathologies—including intracerebral hemorrhage, hypoxic encephalopathy following cardiac arrest, subarachnoid hemorrhage, cerebral edema in the absence of cardiac arrest, and cerebral ischemia—were more frequently reported in the literature cohort. By comparison, subacute findings, particularly nonspecific white matter lesions and spinal cord (myelon) lesions, were more common in our clinical cohort.

Generally, on admission to the emergency department in every patient after LI a cranial CT should be done to rule out ischemia, bleeding and edema, as these pathologies may be without clinical finding and might also deteriorate. One should also be aware of the fact, that many transient neurological symptoms, including paresis, sensory deficits and dysarthria have been reported without any finding in cranial CT or MRI [12, 22]. If cranial CT has been normal, but the patient has persisting neurological symptoms, further neuroimaging by cranial MRI as well as electrophysiologic workup might be needed. Given reasonable clinical suspicion, the additional acquisition of a spinal MRI is suggested. In our experience it might additionally be advisable to do brain and spinal MRI in persons who had a LI during their professional activity, since occupational disability might occur and a baseline MRI is useful for later legal claims.

Unspecific lesions or lesions that are diagnosed years after the injury should not be directly linked to the lightning prematurely. As in patients without LI, thorough diagnostic workup should be done to rule out autoimmune, infectious, ischemic, toxic-metabolic and other causes of lesions found in neuroimaging.

The retrospective design and the limited number of patients represent limitations of our study. LI is a rare condition and neurologists see only a small number of patients. Since most patients presented to our specialized clinic months or even years after the LI, seeking evaluation for potential neurological sequelae of LI, there is an increased interval between the acute injury and first presentation as well as first neuroimaging. Therefore, our results do not reflect neuroimaging in the acute emergency department situation. Additionally, our study lacks a systematic approach to neuroimaging at specified intervals, which means that temporal changes after LI could not be detected. Although the study predominantly included young patients, who are less likely to have concomitant CNS diseases, the coincident findings in neuroimaging do not imply a causative link to the LI [23]. In our group of patients with unspecific white matter lesions in MRI, 36% had known cardiovascular risk factors, possibly contributing to this pathology before LI. In the remaining cases with pathological findings in neuroimaging, after careful medical history, neurological examination and further diagnostic tests (e.g. lumbar puncture, neurophysiology), we could not find any sign of other underlying neurological diseases. However, alternative causes of the neuroimaging findings were not systematically excluded in every case. Regarding the literature research, there might be a publication bias for patients with lesions on neuroimaging, leading to an overestimation of neuroimaging abnormalities after LI.

## Conclusion

In summary, imaging of the brain and spinal cord after LI reveals different types of lesions, however, no pathognomonic finding. Not every case exhibits clinical correlates of pathological imaging or corresponding CNS lesions for neurological symptoms. Further research is necessary to better understand clinical significance, frequency and temporal development of pathological neuroimaging after LI.

## Supplementary Information

Additional file1 (DOCX 47 KB)

## Data Availability

Anonymized data that has not been published within this article will be made available upon request to any qualified investigator.
