# Supplementary material for: Neurological and neuroimaging sequelae of lightning injury: a retrospective study and literature review
Source: Neurol Res Pract. 2026 Sep 14;8(1):77. doi: 10.1186/s42466-026-00523-2 (PMC13576227; doi:10.1186/s42466-026-00523-2)
Supplement: Supplementary file 1 [file 42466_2026_523_MOESM1_ESM.docx]

*Title:*

Neurological and neuroimaging sequelae of lightning injury: a retrospective study and literature review

*Authors:*

Tim Steinberg^1,2^, Carsten Lukas^3^, Berthold Schalke^1^, Christina Wendl^4^, Ralf Linker^1^, Bernhard Neumann^1,5^, Ingo Kleiter^6,7^

*Affiliations:*

^1^ Department of Neurology, University Medical Center Regensburg, Regensburg, Germany

^2^ Department of Neurology and Neurophysiology, University Medical Center Augsburg, Augsburg, Germany

^3^ Institute of Neuroradiology, St. Josef-Hospital, Ruhr University Bochum, Bochum, Germany

^4^ Department of Neuroradiology, University Medical Center Regensburg, Regensburg, Germany

^5^ Department of Neurology, Donau-Isar-Clinic Deggendorf, Medical Campus Niederbayern, Deggendorf, Germany

^6^ Department of Neurology, St. Josef-Hospital, Ruhr University Bochum, Bochum, Germany

^7^ Marianne-Strauß Klinik Berg, Germany

| **Case number** | **Age at the time of lightning injury** | **Sex** | **Location/activity at the time of injury** | **Type of injury** | **Imaging modality** | **Time of imaging in relation to injury** | **Radiological findings** | **Neurologic examination** | **Reference** |
| --- | --- | --- | --- | --- | --- | --- | --- | --- | --- |
| 1 | 45 | M | riding a bicycle | b | spinal X-Ray  cCT  cCT  cCT | initially  initially  12 hours  3 days | spinal X-Ray: normal  initial cCT: normal  cCT 12 hours: two ICBs on the right convexity, mild SAB, mild cerebral edema  cCT 3 days: bilateral edema, thrombosis of superior sagittal sinus | initially: GCS 3, apnoeic until arrival of paramedics, no reaction to stimuli, Babinski's sign positive on both sides  12 hours: patient ventilated, slight movements of all extremities after painful stimuli  24 hours: patient ventilated, hemiparesis on the left side, Babinski's sign positive on both sides  3 days: persistent loss of consciousness, no reaction to painful stimuli, brain stem reflexes preserved, flaccid tetraparesis  17 days: reacting to verbal commands, moving all limbs | L. B. th, M.D., Successful Management of an Adult Lightning Victim Using Intracranial Pressure Monitoring, *Neurosurgery*, Volume 28, Issue 6, June 1991, Pages 907–910 |
| 2 | 19 | M | standing next to a motor cycle | NA | cCT | 14 months | dilatation of inner ventricular system, possibly due to hydrocephalus e vacuo, generalized atrophy | Initially: GCS 3, apnoeic, cyanotic, later tachypnoeic, blood pressure 240mmHg systolic  Later: acute respiratory distress syndrome and sympathicotonic autonomous dysfunction, flaccid tetraparesis  14 months: full recovery | Hawkes, C. H. und Thorpe, J. W. (1992). Acute polyneuropathy due to lightning injury. J Neurol Neurosurg Psychiatry 55(5), 388-390 |
| 3 | 21 | M | working outdoors | NA | spinal and cerebral X-Ray  cCT | initially | all normal | initially: CPR, tetraplegia at C4 level, no sensory loss  later: persistent tetraplegia at C5 level, no sensory loss | Cherington, M., Yarnell P. und Lammereste, D. (1992). Lightning strikes: nature of neurological damage in patients evaluated in hospital emergency departments. Ann Emerg Med. 21(5), 575-578 |
| 4 | 16 | M | camping | b | spinal X-ray  cCT | initially | all normal | initially: CPR, sensomotoric paraplegia, transient aphasia  6 weeks: persistent paraplegia, no more aphasia or sensory loss | Cherington, M., Yarnell P. und Lammereste, D. (1992). Lightning strikes: nature of neurological damage in patients evaluated in hospital emergency departments. Ann Emerg Med. 21(5), 575-578 |
| 5 | 15 | M | hiking | NA | cMRI | 1 day | posterior parietal ischemia on the right side | initially: hemiplegia on the left  three years: minor paresthesias of the left leg, no paresis | Cherington, M., Yarnell P. und Lammereste, D. (1992). Lightning strikes: nature of neurological damage in patients evaluated in hospital emergency departments. Ann Emerg Med. 21(5), 575-578 |
| 6 | 38 | M | skiing | NA | cCT  cMRI | initially  2 days | cCT initially: diffuse hypoxic encephalopathy  cMRI 2 days: diffuse hypoxic encephalopathy, hyperintense lesions of basal ganglia and grey matter, narrowing of subarachnoid space and ventricles | initially: CPR, persistent loss of consciousness, death after 4 days | Cherington, M., Yarnell, P. und Hallmark, D. (1993). MRI in lightning encephalopathy. Neurology 43(7), 1437-1438 |
| 7 | 64 | F | riding a horse | NA | cCT  cMRI | 7 days | all normal | initially: CPR 1 week: atactic gait, truncal ataxia, atactic finger-to-nose test | Cherington, M., Yarnell, P. und Hallmark, D. (1993). MRI in lightning encephalopathy. Neurology 43(7), 1437-1438 |
| 8 | 33 | M | camping | NA | cMRI | 7 days | cerebellar atrophy, fluid accumulation in the mastoid part of the right temporal bone | initially: numbness and stiffness of the limbs, perforated right tympanum  1 week: atactic finger-to-nose test  later: persistent ataxia | Cherington, M., Yarnell, P. und Hallmark, D. (1993). MRI in lightning encephalopathy. Neurology 43(7), 1437-1438 |
| 9 | 57 | M | sitting in a golf cart | NA | cCT | initially | hypoxic encephalopathy | initially: unconsciousness, ventricular fibrillation  later: no reaction to stimuli, myoclonia of all extremities, death after 3 days | Cherington, M., Yarnell, P. R. und London, S. F. (1995). Neurologic Complications of Lightning Injuries. West J Med. 162, 413-441 |
| 10 | 30 | M | walking through glass door | NA | cMRI | 4 days | normal | 4 days: facial palsy on the right  3 weeks: incomplete recovery | Cherington, M., Yarnell, P. R. und London, S. F. (1995). Neurologic Complications of Lightning Injuries. West J Med. 162, 413-441 |
| 11 | 32 | M | on a boat | NA | cCT | initially | ICB in the basal ganglia on both sides | initially: immediate CPR with ROSC after 30 seconds  admission: lethargic, following commands, no paresis, atactic gait, combined sensory and conductive hearing loss  8 months: deafness, otherwise recovered | Ozgun B, [Castillo M](https://www.ncbi.nlm.nih.gov/pubmed/?term=Castillo%20M%5BAuthor%5D&cauthor=true&cauthor_uid=7677044), Basal ganglia hemorrhage related to lightning strike. AJNR 1995 Jun-Jul;16(6):1370-1 |
| 12 | 46 | M | working on mobile storm tracking unit | NA | cMRI | 5 days | multiple juxtacortical supratentorial hyperintense lesions | initially: no loss of consciousness, pain in the left arm and paresis of the left leg  8 days: atactic broad-based gait, slurred speech, neuropsychological deficits  later: neuropsychological deficits, intermittent episodes of confusion | Milton, W. J., Hal O'Dell, R. und Warner, E. G. (1996). MRI of lightning injury: early white matter changes associated with cerebral dysfunction. J Okla State Med Assoc. 89(3), 93-94 |
| 13 | 51 | M | standing beside a bicycle | a | cCT  cMRI  c/sMRI | initially  9 days  19 days | all normal | initially: CPR with ROSC after 19 minutes, positive Babinski's sign on both sides  6 days: eye focusing possible  9 days: following commands  following months: able to sit, atactic gait, ataxia of upper extremities, apraxia, neuropsychological deficits, mood instability, intermittent confusion  19 months: poor initiation of actions, psychomotor slowing, neuropsychological deficits, living in a care facility | Cherington, M., Krider, E. P., Yarnell, P. R. und Breed, D. W. (1997). A bolt from the blue: lightning strike to the head. Neurology 48(3), 683-686 |
| 14 | 18 | M | standing on a forest road | a | cCT  cMRI | 8 days | cCT: perithalamic ICB in the left hemisphere  cMRI: lesion of the left putamen | initially: immediate CPR,  admission: coma, focal epileptic seizure  8 days: opening eyes, no motor reaction, loss of deep tendon reflexes on the right  18 months: residual neuropsychological deficits, fatigue, mood instability | Van Zomeren, A. H., ten Duis, H. J., Minderhoud, J. M. und Sipma, M. (1998). Lightning stroke and neuropsychological impairment: cases and questions. J Neurol Neurosurg Psychiatry 64, 763-770 |
| 15 | 26 | M | working in the garden | NA | cranial x-ray  cMRI | initially  few days | all normal | initially: loss of consciousness for 10 minutes, afterwards hyperactive delirium  later: acute stress reaction with psychosis, headache | Tan, H. H. und Goh, S. H. (2003). Lightning injury: Changi Hospital experience. Hong Kong J. emerg. med. 10(4), 223-232 |
| 16 | 13 | M | standing on playground at school | a | cCT | initially | normal | initially: CPR, duration unknown  admission: GCS 3, hemiparesis on the right side, later following commands  day 4: Moderate hemiparesis on the right side, expressive dysphasia | Courtman, S. P., Wilson, P. M. und Mok, Q. (2003). Case report of a 13-year-old struck by lightning. Paediatr Anaesth. 13(1), 76-79 |
| 17 | 58 | M | sleeping in a tent | NA | sMRI  sMRI | 6 weeks  12 weeks | sMRI 6 weeks: T2 and STIR hyperintense lesions in posterolateral column C1-C3 on both sides  sMRI 12 weeks: no more hyperintensities | initially: short loss of consciousness, keraunoparalysis  2 days: gait uncertainty, otherwise normal  6 weeks: progressive numbness, tingling, dysesthesias in both arms and feet, weakness of intrinsic muscles of both hands, feeling of shocks going through trunk, arms and feet elicit by neck flexion, positive Lhermitte's sign,  12 weeks: partial recovery of the above-mentioned symptoms | Freeman, C. B., Goyal, M. und Bourque, P. R. (2004). MR imaging findings in delayed reversible myelopathy from lightning strike. Am J Neuroradiol. 25(5), 851-853 |
| 18 | 45 | M | NA | NA | cCT  cCT  cMRI | initially  3 days  4 days | initial cCT: mild brain edema cCT 3 days: no change  cMRI 4 days: infarction predominantly in the right parietal, temporal, and frontal lobes and cerebellar on both sides | initially: GCS 13, dysarthria, bilaterally ruptured tympanic membranes  4 days: walking with help, ataxia  1 month: persistent ataxia | Aslan, S., Yilmaz, S. und Karcioglu, O. (2004). Lightning: an unusual cause of cerebellar infarction. Emerg Med J. 21(6), 750-751 |
| 19 | 24 | M | sitting on car | b | cMRI | 7 years | normal | initially: transient loss of consciousness, confusion, later normal  1 year: diminished attention and concentration  2 years: social distancing, irritability, panic attacks, headache, acoustic hallucinations, depression | Reisner, A. D. (2006). A case of lightning injury with delayed-onset psychiatric and cognitive symptoms. Brain Inj. 20(10),1093-1097 |
| 20 | 51 | M | under a tree | b | cCT  cMRI | 3 days  3 days | all normal | Initially: Loss of consciousness, cardiac arrest. CPR on arrival of medical service.  on admission: anisocoria and lens opacity of the right eye  approx. 1 week: after extubation retrograde amnesia, cataract of the right eye.  2-4 weeks: initially unilateral left, later bilateral facial paresis, tingling sensation in all extremities, progressive tetraparesis, generalized hyporeflexia, cytalbuminologic dissociation in lumbar puncture, diagnosis of GBS, IVIG therapy. | Theodorou, P., Limmroth, V., Perbix, W., Gerbershagen, M., Knam F. und Spilker, G. (2008). Guillain-Barré syndrome after lightning strike. Burns, 34(5), 722-726 |
| 21 | 47 | M | getting out of a car | NA | sMRI | few days | normal | initially: distal and proximal paraparesis MRC 4/5, loss of deep tendon reflexes of legs, pallhypesthesia, pin-prick discrimination and hypothermesthesia to the level of L1 bilaterally  2 days: brisk deep tendon reflexes of legs, positive Babinski’s sign on the right side  4 weeks: brisk deep tendon reflexes of legs, positive Babinski’s sign on the right side, mild spasticity in both legs, decreased pin prick sensation and proprioception involving the distal aspect of the right leg | - Lakshminarayanan, S., Chokroverty, S., Eshkar, N. und Grewal, R. (2009). The spinal cord in lightning injury: a report of two cases. J Neurol Sci. 276(1-2), 199-201 |
| 22 | 39 | M | NA | NA | cMRI  cMRI  sMRI  sMRI  cMRI | initially  3 weeks  3 months  4 years  4.6 years | initial cMRI: ICB in the left thalamus and the left basal ganglia  cMRI 3 weeks: ICB in the left thalamus and the left caudate nucleus, residual subdural hematomas in the occipital regions bilaterally and along the right frontal region  sMRI 3 months: diffuse hyperintensity C1-C6  sMRI 4 years: normal  cMRI 4.6 years: periventricular white matter lesions on the left consistent with the initial ICB | initially: loss of consciousness, sensomotoric hemiparesis on the right  3 months: hypoesthesia and paresthesias in all extremities, pallhypesthesia of fingers and toes, moderate spastic hemiparesis on the right MRC 4/5, wide based gait, dysarthria  5 years: improved spastic hemiparesis on the right, no sensory problems | - Lakshminarayanan, S., Chokroverty, S., Eshkar, N. und Grewal, R. (2009). The spinal cord in lightning injury: a report of two cases. J Neurol Sci. 276(1-2), 199-201 |
| 23 | 8 | M | under a tree | b | cCT | initially | generalized brain edema | initially: loss of consciousness, no cardiac arrest at first, death because of arrhythmias 24 hours after injury | Mücahit Emet, Ahmet Kayal, Şahin Aslan, Mehmet Karacan, Gökhan Ersunan. Lightning-induced lethal myocardial infarction and ventricular tachycardia in a boy. Anatol J Cardiol. 2010; 10(4): 378-379 |
| 24 | 9 | F | NA | NA | cCT  several cMRI | initially  few weeks | all normal | initially: loss of consciousness, confusion  later: cerebral salt wasting syndrome lasting 6 months | Orbak, Z., Kara, İ.S. Unusual complication in a child with lightning strike: cerebral salt wasting. *Childs Nerv Syst* **26,** 1125–1127 (2010). |
| 25 | 17 | M | working in the field | NA | cCT | initially | ICB in the lentiform nucleus on the right side with mass effect. CT angiography without abnormalities | initially: loss of consciousness, hemiplegia on the left | Aggarwal, A. und Salunke, P. (2011). Bolt from the blue: Basal ganglion bleed following lightning strike. Surg Neurol Int. 2, 170 |
| 26 | 28 | F | on a field next to a tree, which the lightning hit | c | cCT | initially | normal | initially: transient loss of consciousness, dysarthria, dysphagia, muscle spasms, paresthesias  1 year: normal | Desai BK, Fairclough R. A case of a speech impediment following a near lightning strike. International Journal of Emergency Medicine. 2011 ;4:60. DOI: 10.1186/1865-1380-4-60. |
| 27 | 50 | M | under a tree | NA | cMRI  sMRI | few days | cMRI: normal sMRI: rupture of posterior longitudinal ligament T12-L1 with signs of hemorrhage | initially: transient loss of consciousness 15 minutes, sensomotoric keraunoparalysis to the T12 level, urinary retention  following days: improved strength | Kumar, A., Srinivas, V. und Sahu, B. P. (2012). Keraunoparalysis: What a neurosurgeon should know about it? J Craniovertebr Junction Spine 3(1), 3-6 |
| 28 | 10 | M | under a tree | a | X-ray  cMRI | few days  2 weeks | X-ray: normal  cMRI 2 weeks: Chiari malformation I, syringomyelia | initially: transient loss of consciousness, afterwards no complaints  2 weeks: neck pain | Tubbs, R.S., Muhleman, M., Loukas, M. *et al.* Chiari I malformation and syringomyelia in a child following a lightning strike. *Childs Nerv Syst* **28,** 751–753 (2012). |
| 29 | 24 | M | riding a motor cycle | NA | cCT  cCT  cMRI | initially  14 days  20 days | initial cCT: traumatic SAB and cerebral edema without mass effect cCT 14 days: sinusitis cMRI 20 days: severe hypoxic encephalopathy | initially: cardiac arrest for 5min, CPR  admission: GCS 3, later facial myoclonia and generalized convulsions,  following weeks: no reaction to painful stimuli, preserved brain stem reflexes, death after 84 days | Forster, S.A., Silva, I.M., Ramos, M.L.C., Gragnani, A., Ferreira, L.M. (2013). Lightning burn - review and case report. Burns 39(2), 8-12 |
| 30 | 24 | M | connecting a trailer to a car | d | cMRI  sMRI | few months | cMRI: unspecific hyperintense lesions sMRI: disc protrusion C7-T1 without spinal cord compression | initially: confusion, later normal  3,5 months: posttraumatic stress disorder with functionally impaired fine motor skills and sensory problems, central hyperadrenergic state with action tremor of the hands, tachycardia, hypertension | Parsaik, A. K., Ahlskog, J. E., Singer, W., Gelfman, R., Sheldon, S. H., Seime, R. J., Craft, J. M., Staab, J. P., Kantor, B. und Low, P. A. (2013). Central hyperadrenergic state after lightning strike. Clin Auton Res. 23(4), 169-173 |
| 31 | 29 | M | standing on a tractor | NA | cMRI  cMRI | 4 days  8 days | cMRI 4 days: FLAIR-hyperintensities in paramedian thalamus, hippocampus, dorsolateral globus pallidus and proximal medulla oblongata on both sides. Restricted diffusion with hypointense ADC-correlate in gyrus praecentralis, corona radiata along the corticospinal tract. Diffusion restriction in the cerebellum on both sides. Imaging interpreted as consistent with hypoxic brain damage.  cMRI 8 days: no change | initially: loss of consciousness, cardiac arrest, CPR and ROSC after 35min.  1 day: Only voluntary efforts: vertical eye movements and blinking, flaccid tetraplegia, corneal reflex and oculocephalic reflex absent.  7 days: tetraplegia, communicating via blinking. slight facial movement on the left as reaction to pain on the left finger  6 months: able to abduct right arm about 20°, to nod and shake the head for communication and to move the tongue, permanently dependent in activities of daily living. | Abdulla, S., Conrad, A., Schwemm, K. P., Stienstra, M. P., Gorsselink, E. L., Dengler, R. und Abdulla, W. (2014). Lesions along the upper motor neuronal pathway with locked-in features after lightning strike and cardiac arrest: a case-review analysis. Brain Inj. 28(3),. 298-303 |
| 32 | 53 | M | working in the field | NA | cMRI  sMRI | 6 weeks | all normal | initially: loss of consciousness for 3 hours, after that severe pain and swelling of the right arm  6 weeks: distal paresis of the right arm MRC 2/5, sensory loss in the segments C5-C7. Diagnosed as brachial plexopathy | Bhargava, A. N., Kasundra, G. M., Khichar S. und Bhushan, B. S. (2014). Lightning strike-  induced brachial plexopathy. J Neurosci Rural Pract. 5(4), 399-400 |
| 33 | 22 | M | mountaineering | NA | cCT  cMRI  sMRI | initially | all normal | initially: sensomotoric hemiparesis (keraunoparalysis) on the left side MRC 1/5  1 day: residual paresthesias in the affected areas, otherwise full recovery | Rahmani, S. H., Faridaalaee, G. und Jahangard, S. (2015). Acute transient hemiparesis induced by lightning strike. Am J Emerg Med. 33(7), 984 |
| 34 | 19 | M | riding a motorcycle | a | MRI neck | approx. 1 week | normal | initially: flaccid sensomotoric paresis of the left arm  approx. 1 week: persistent paresis, neurophysiologic examination suggestive for neurapraxia, therapy with methylprednisolone  1 month: full recovery | Senthilkumaran, S., Balamurugan, N., Jayaraman, S., Sasikumar, S. und Thirumalaikolundusubramanian, P. (2015). Peripheral nervous system involvement in lightning strike-the devil in disguise. Am J Emerg Med. 33(11), 1704-1705 |
| 35 | 25 | M | holding a telephone pole | e | MRI neck | few days | normal | initially: sensomotoric flaccid paresis of the right arm MRC 0/5  4 weeks: full recovery | Patnaik, A., Mahapatra, A. K. und Jha, M. (2015). Pan-brachial plexus neuropraxia following lightning: A rare case report. Surg Neurol Int. 19(6), 110-112 |
| 36 | 19 | F | NA | NA | cMRI | approx. 2 weeks | normal | bilateral sensory hearing loss | Turan M, Kalkan F, Bozan N, et al. Isolated Sensorineural Hearing Loss as a Sequela after Lightning Strike. *Case Rep Otolaryngol*. 2015;2015:738416. |
| 37 | 75 | M | NA | NA | sMRI | initially | normal | initially: loss of consciousness  admission: paraplegia, hypoesthesia up to the T12 level, loss of anal reflex and arbitrary sphincter tone  1 year: no complaints | Mohamad Gouse, Justin Arockiaraj, Ronald Khanapur, and Gangadaran Srinivasan. Transient paraplegia in an elderly due to lightning injury: An unusual cause. J Emerg Trauma Shock. 2015 Oct-Dec; 8(4): 238–239. |
| 38 | 20 | M | NA | a | cCT  cCT  cCT  cCT | initially  2 days  6 days  8 days | initial: hematotympanum  cCT 2 days: cerebellar infarction  cCT 6 days: generalized brain edema, bilateral cerebellar infarction with mass effect and herniation  cCT 8 days: progressive herniation in spite of occipital trepanation | initially: loss of consciousness, CPR  8 days: coma, loss of brain stem reflexes, loss of SEPs on both sides, death | Gruhn, K. M., Knossalla, F., Schwenkreis, P., Hamsen, U., Schildhauer, T. A., Tegenthoff, M. und Sczesny-Kaiser, M. (2016). Neurologische Erkrankungen nach Blitzschlag. Der Nervenarzt 87(6), 623-628 |
| 39 | 20 | M | playing soccer | a | cMRI  sMRI | approx. 5 weeks | all normal | initially: CPR  5 weeks: flaccid tetraparesis, loss of deep tendon reflexes, hypesthesia in both legs, dysarthria, organic mental disorder, diagnosis of chronic inflammatory demyelinating polyneuropathy  after rehabilitation: no paresis, mild organic mental disorder | Gruhn, K. M., Knossalla, F., Schwenkreis, P., Hamsen, U., Schildhauer, T. A., Tegenthoff, M. und Sczesny-Kaiser, M. (2016). Neurologische Erkrankungen nach Blitzschlag. Der Nervenarzt 87(6), 623-628 |
| 40 | 37th week of gestation | F | mother hit by lightning while watching a fireworks show | a or c | cMRI | 1 day | bilateral symmetric ischemia involving posterior limbs of internal capsule | Initially: mother with minor complaints, cesarean section due to fetal tachycardia and reduced variability. After birth periodic shaking, EEG showing burst suppression and diffuse cerebral dysfunction.  2 weeks: EEG slightly improved, diagnosis of diffuse cortical injury.  1 year: unable to sit, crawl or swallow. | [Galster K](https://www.ncbi.nlm.nih.gov/pubmed/?term=Galster%20K%5BAuthor%5D&cauthor=true&cauthor_uid=27116922)^1^, [Hodnick R](https://www.ncbi.nlm.nih.gov/pubmed/?term=Hodnick%20R%5BAuthor%5D&cauthor=true&cauthor_uid=27116922)^2^, [Berkeley RP](https://www.ncbi.nlm.nih.gov/pubmed/?term=Berkeley%20RP%5BAuthor%5D&cauthor=true&cauthor_uid=27116922)^1^ Lightning Strike in Pregnancy With Fetal Injury.  [Wilderness Environ Med.](https://www.ncbi.nlm.nih.gov/pubmed/27116922) 2016 Jun;27(2):287-90. |
| 41 | 7M | F | lying in a baby cradle close to a window | NA | cMRI  cMRI | 8.5 years  9.5 years | cMRI 8.5 years: diffuse white matter lesions only in the right hemisphere  cMRI 9.5 years: no change | initially: no obvious complaints  8.5 years: bifrontal headaches with nausea, mother with known migraine | IVJ, Kuqo A, Grabova S, Rroji A, Vyshka G. Right Hemispheric Leukoencephalopathy as an Incidental Finding Following a Lightning Strike. Open Access Maced J Med Sci. 2016 Dec 15; 4(4):692-694. |
| 42 | 75 | F | standing next to a tree | b | cCT  sMRI | initially  2 weeks | cCT: normal  sMRI 2 weeks: old fracture of vertebral body T2 | initially: retrograde amnesia  2 weeks: dysesthesia in the L5 dermatome on the right side, allodynia and numbness in both legs, gait uncertainty  6 weeks: no change | Tadler M, Rüegg E, Niquille M, et al. Multi-organ injuries due to a lightning strike: a case report highlighting the importance of a multi-disciplinary approach. *Case Reports Plast Surg Hand Surg*. 2017;4(1):1‐4. Published 2017 Jan 6. |
| 43 | 40 | M | standing on a parking area, holding an umbrella | a | cCT  CT neck | initially | all normal | initially: transient loss of consciousness, paraparesis, hypesthesia of the left leg.  admission: diffuse pain, otherwise no complaints  later: no complaints | Eyerly-Webb SA, Solomon R, Lee SK, et al. Lightning Burns and Electrical Trauma in a Couple Simultaneously Struck by Lightning. *Clin Pract Cases Emerg Med*. 2017;1(3):246‐250. Published 2017 Jul 17. |
| 44 | 30 | F | standing on parking area, holding an umbrella | a | cCT  CT neck | initially | all normal | no complaints | Eyerly-Webb SA, Solomon R, Lee SK, et al. Lightning Burns and Electrical Trauma in a Couple Simultaneously Struck by Lightning. *Clin Pract Cases Emerg Med*. 2017;1(3):246‐250. Published 2017 Jul 17. |
| 45 | 5 | M | next to father who was hit by a lightning | b | cCT  sMRI | initially | all normal | initially: seizure  6 days: normal | Aseel Sleiwah et al: Lightning injuries in Northern Ireland. *Ulster Med J* 2018;**87(3):**168-172. |
| 46 | 36 | M | NA | a | cCT  sCT  cMRI  sMRI | day 6  day 6  not specified | cCT 6 days: left temporal infarction, small SAB, loss of grey-white matter differentiation  sCT 6 days: normal  cMRI: hypoxic encephalopathy, left temporal infarction  sMRI: normal | initially: CPR with ROSC after 50min  later: generalized weakness, confusion, neurography showing peripheral axonal sensomotoric neuropathy  after rehabilitation: tetraparesis | Aseel Sleiwah et al: Lightning injuries in Northern Ireland. *Ulster Med J* 2018;**87(3):**168-172. |
| 47 | 51 | F | inside the house | NA | cCT | initially | normal | initially: hemiparesis on the right side  on admission: sensomotoric hemiparesis on the right side, hypoesthesia of the right face  2 weeks: slight hypoesthesia on the right side, dizziness | Sadananda B. Naik, R. V. Murali Krishna:  A Case of Keraunoparalysis: A Bolt from the Blue  Indian J Crit Care Med. 2018 Nov; 22(11): 804–805. |
| 48 | 19 | M | NA | NA | cCT | initially | normal | initially: retrograde amnesia, disorientation concerning time.  6 months: no complaints | Uzel Şener M, Demir A, Şener A. Lightning-strike-induced acute lung injury: a case report. Ulus Travma Acil Cerrahi Derg 2019;25:198-201. |
| 49 | 6 | F | standing next to power pole | b | cCT | initially | mild brain edema | Initially: emesis, convulsions without loss of consciousness.  later: no complaints | Thasneem, K., Ratageri, V.H. & Fattepur, S.R. Lightning Injury in Children. *Indian J Pediatr* **86,** 969 (2019). |
| 50 | 45 | M | attached to a metal chain while hiking in the mountains | d | cCT | initially | normal | neuropraxia of the right peroneal nerve and bilateral tinnitus | Kosiński S, Aleksandrowicz D, Serafinowicz Z. A Storm, An Explosion, and Flying Rocks - An Unusual Injury due to a Lightning Strike in the Mountains. *Prehospital and Disaster Medicine*. 2022;37(4):547-549. |
| 51 | 19 | M | participating in outdoor military activity | c | cCT, CTA | within the first days of treatment | normal | initially: altered mental status  Later: headache, otherwise no complaints | Kruchevsky D, Sadeh O, Dominsky O, Levanon S, Ramon I, Ullmann Y. Case Report: Burns Through Metal - Unique Pattern Of First Lightning Strike Injury In Israeli Army. Ann Burns Fire Disasters. 2020 Jun 30;33(2):170-173. |
| 52 | 50 | M | riding a bicycle in the forest | a | cCT  cCT  cCT, CTA | initially  day 2  day 5 | cCT initially: right apical and temporal subarachnoid hemorrhage, millimeter-sized petechial hemorrhages in the cortical- subcortical junction of the fronto-parietal lobes, and slight signs of initial edema  cCT 2 days: reduction of the cortico-subcortical differentiation bilaterally in the frontal-parietal and temporo-occipital areas compatible with anoxic edema, a marked flattening of the cortical convolutions, and the obliteration of the supratentorial peri-encephalic liquor spaces, also at the level of basal cisterns  cCT, CTA 5 days: CTA normal, cCT with further reduction of cortico-subcortical differentiation | initially: CPR with ROSC after 16min. GCS 3.  Day 3: GCS 3, absence of pupillary and corneal reflexes  Day 5: GCS 3, weak cough reflex and respiratory drive | Roman-Pognuz E, Moro E, Macchini E, Di Paolo E, Pesenti K, Lucangelo U, Bussani R, Baratella E, Pellis T, Ristagno G. Neuronal desertification after a direct lightning strike: a case report. J Med Case Rep. 2022 Oct 21;16(1):391. |
| 53 | 38 | M | performing aircraft maintenance on an airport, seeking shelter under the wing of an aircraft | a | cCT | initially | normal | Initially: generalized tonic-clonic seizure 30 seconds. Afterwards GCS 9, paresthesias of the legs  Day 3: normal | Chlouchi A, Hmadate I, Chafi K, Rafai M, Outaghyame O, Benabbouha A, Bazine A, Elhjouji A. Seizure, A Shocking Effect of a Lightning Strike on Aerodrome: A Case Report. Air Med J. 2024 Mar-Apr;43(2):168-170. |
| 54 | 6 | M | storm chasing | a | cCT, CT cervical spine | initially | normal | Initially: confused, muscle cramps  10 weeks: memory disturbances, anxiety | Shipman J, Carver B, Painter K, Shipman S. The Dangerous Life of a Storm Chaser: A Lightning Strike Injury Causing Serious Injury. J Investig Med High Impact Case Rep. 2020 Jan-Dec;8:2324709620925566. |

**Supplementary Table 1: Overview of literature cases describing neurological findings and imaging of the neuroaxis after lightning injuries 1990 – 2024.** a = direct strike, b = side splash, c = ground current, d = contact voltage, e = wire mediated. APGAR score, appearance, pulse, grimace, activity and respiration score; cCT, cranial computed tomography; CPR, cardiopulmonary resuscitation; F, female; GCS, Glasgow coma scale; ICB, intracerebral bleeding; M, male; MRC, medical research council; NA, not available, meaning information not specified in the cited article; ROSC, return of spontaneous circulation; SAB, subarachnoidal bleeding, SEP, sensory evoked potentials; sCT, spinal computes tomography; cMRI, cranial magnetic resonance imaging; sMRI, spinal magnetic resonance imaging.
